# P-2352. Post-pandemic SARS-CoV-2 vaccination and infection status and risk of influenza-like illnesses and work absenteeism in healthcare workers – a prospective cohort study

**DOI:** 10.1093/ofid/ofae631.2504

**Published:** 2025-01-29

**Authors:** Tamara K Dörr, Joanne Lacy, Tala Ballouz, Alexia Cusini, Fabian Grässli, Sarah Haile, Emina Kocan, J Carsten Möller, Milo Alan Puhan, Matthias Schlegel, Matthias Von Kietzell, Markus Rütti, Reto Stocker, Danielle Vuichard-Gysin, Christian Kahlert, Stefan Kuster, Philipp Kohler

**Affiliations:** Cantonal Hospital St. Gallen, St. Gallen, Sankt Gallen, Switzerland; University of Zurich, Zurich, Zurich, Switzerland; University of Zurich, Zurich, Zurich, Switzerland; Cantonal Hospital Graubünden, Chur, Graubunden, Switzerland; Cantonal Hospital St. Gallen, St. Gallen, Sankt Gallen, Switzerland; University of Zurich, Zurich, Zurich, Switzerland; Geriatric Clinic St. Gallen, St. Gallen, Sankt Gallen, Switzerland; Center for Neurological Rehabilitation, Zihlschlacht, Thurgau, Switzerland; University of Zurich, Zurich, Zurich, Switzerland; Cantonal Hospital St. Gallen, St. Gallen, Sankt Gallen, Switzerland; Hirslanden Clinic Stephanshorn, St. Gallen, Sankt Gallen, Switzerland; Fuerstenland Toggenburg Hospital Group, Wil, Sankt Gallen, Switzerland; Hirslanden Clinic Zurich, Zurich, Zurich, Switzerland; Thurgau Hospital Group, Muensterlingen, Thurgau, Switzerland; Cantonal Hospital St. Gallen, St. Gallen, Sankt Gallen, Switzerland; Cantonal Hospital St. Gallen, St. Gallen, Sankt Gallen, Switzerland; Hopital St. Gallen, St. Gallen, Zurich, Switzerland

## Abstract

**Background:**

In the transition to SARS-CoV-2 endemicity, we prospectively assessed whether the number of SARS-CoV-2 vaccinations was associated with influenza-like illness (ILI) episodes or work absenteeism in a cohort of healthcare workers (HCW).

**Figure:**
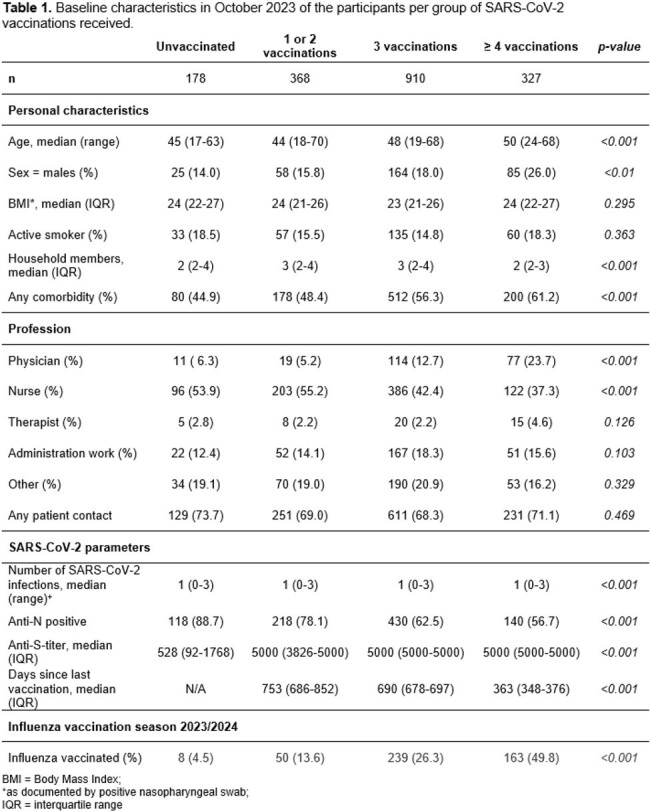

**Methods:**

In October 2023, we updated information on SARS-CoV-2 vaccination and infection within a prospective multi-centre HCW cohort. Anthropometric data and information on personal health, patient contact, and household structure were collected. Between 11/2023 and 04/2024 (i.e. follow-up), where community activity of SARS-CoV-2 and influenza were high, HCW filled in weekly questionnaires on acute viral symptoms and number of days absent from work. The main predictor was SARS-CoV-2 vaccination status grouped into unvaccinated, 1-2, 3, or≥ 4 vaccine doses. Outcomes were the number of ILI episodes and days absent from work. ILI was defined as acute-onset (≤ 7 days) of fever/feverish feeling and one respiratory symptom (runny nose, loss of smell, cough, sore throat). Using a negative binomial model, we calculated adjusted incidence rate ratios (aIRR) and 95% confidence intervals (CI) for SARS-CoV-2 vaccination status and the outcomes, adjusting for important confounders.

**Figure:**
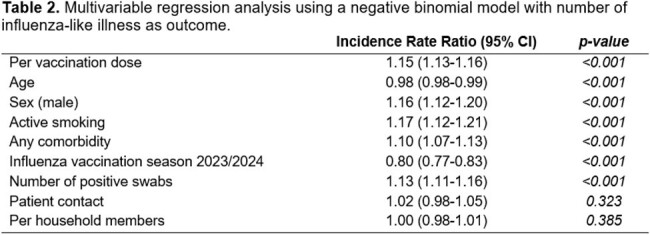

**Results:**

We included 1’783 HCW (median 47 years, range 17-70), 178 (9.9%) were unvaccinated, 368 (20.6%) had 1-2 vaccinations, 910 (51.0%) 3 vaccinations and 327 (18.3%) ≥4 vaccinations (Table 1). Overall, 764 (42.8%) experienced ≥ 1 ILI episode during follow-up, the mean number of days absent from work was 2. In multivariable analysis, number of SARS-CoV-2 vaccinations was positively associated with number of ILI episodes (aIRR per dose 1.15, 95% CI 1.13-1.16), as did the number of positive SARS-CoV-2 tests (aIRR 1.13, 95% CI 1.11-1.16). Seasonal influenza vaccination (aIRR 0.80, 95% CI 0.77-0.83) was associated with decreased risk (Table 2). SARS-CoV-2 vaccination was also associated with days absent from work (aIRR 1.08, 95% CI 1.06-1.10).

**Conclusion:**

The number of vaccinations against SARS-CoV-2 is not associated with decreased ILI episodes and absenteeism in HCW during high community transmission level of SARS-CoV-2 and influenza. In contrast, smoking and male sex are associated with increased and influenza vaccination with decreased risk.

**Disclosures:**

Tala Ballouz, MD PhD, Moderna: Grant/Research Support

